# The Rhesus Factors—II: Haemolytic Disease of the New-Born

**Published:** 1947

**Authors:** Beryl D. Corner

**Affiliations:** Honorary Physician to Out-Patients, Bristol Children's Hospital. Pædiatrician, Southmead Municipal Hospital


### THE RHESUS FACTORS—II

### Haemolytic Disease of the New-Born

BY

Dr. Beryl D. Corner, M.D., B.S., M.R.C.P.

*Honorary Physician to Out-Patients, Bristol Children's Hospital.*
*Pœdiatrician, Southmead Municipal Hospital.*

---

Diamond and Blackfan (1932) first grouped together three obscure syndromes of the new-born under the name of Erythroblastosis Foetalis, showing that these conditions were all characterized by the presence of large numbers of primitive red cells, erythroblasts, in the circulating blood of the infant. Hydrops Foetalis is the severest form of the disease, the infant being frequently still-born or only surviving for a few hours after birth. There is gross generalized oedema with fluid in the serous cavities, enlargement of the liver and spleen and severe anaemia. The placenta is usually very large and oedematous. This condition is known to occur very rarely in all mammals with long gestation periods, and is termed " water-belly " in elephants. Icterus Gravis Neonatorum is the commonest of these clinical conditions. It has long been recognized as a familial disease, often not affecting the first child, and tending to become worse with each succeeding pregnancy. The amniotic fluid and vernix are often bright yellow, the infant is jaundiced at birth or shortly after; jaundice deepens rapidly and is accompanied by extreme lethargy, with enlargement of liver and spleen, and ensuing severe anaemia if the infant survives the first few days. Hampson (1928) reported a series of these cases with 80 per cent. mortality rate. The surviving infants were frequently found to have cirrhosis of the liver and neurological signs of basal ganglia damage, termed kernicterus. Congenital Anaemia is a rarer manifestation, with a higher survival rate. The anaemia, which is severe, is frequently not recognized until the end of the first week and is often accompanied by little jaundice. It is now known that otherwise unexplained still-birth may also be a manifestation of this disease.

*Theories of Causation.*—For many yeaıs spasmodic interest has been evoked in morbid reactions taking place between the mother and her foetus. Dienst (1908) suggested that "the mother is immunized by the blood of her foetus which might cause toxaemia of pregnancy," and Ottenberg (1923) suggested that icterus gravis neonatorum was due to accidental placental transfusion of incompatible blood.

Diamond and Blackfan, however, laying stress on the presence of erythroblastosis in the infant, thought the abnormality lay in blood formation rather than destruction, and their work stimulated some interest in this condition during the ensuing years. A certain number of patients, chiefly with severe anaemia, survived long enough to find their way to Children's Hospitals, and a certain number of babies with severe jaundice died in Maternity Hospitals during the first few days of life. The treatment given was intramuscular injection of blood from either parent, or an intravenous transfusion, usually Group O, but often given without grouping. The mortality rate was 50–60 per cent. in various reported series from Children's Hospitals. In 1934 it was again suggested that a haemolytic agent was the cause (Hawksley and Lightwood).

The discovery of the Rh factor by Wiener in 1940, and the suggestion by Levine and others in 1941 that Erythroblastosis Foetalis was due to immunization of the mother by the Rhesus antigen from her foetus, or previous blood transfusion, opened the door to a fascinating new field of work on this hitherto obscure disease. It also showed that the condition was of considerable importance in that it occurred more frequently than had been previously believed, and, in fact, is probably the most important single cause of congenital abnormality. The condition was re-named Haemolytic Disease of the New-born. Discovery has advanced rapidly since 1941 and we now believe that this antigen-antibody reaction takes place not only in the infant's red cells, but also in many body tissues, particularly those of the blood-forming organs—liver and spleen (Baar).

*Clinical Diagnosis.*—Modern knowledge of the treatment of this disease makes early diagnosis essential. Wherever possible an attempt should be made to forecast the likelihood of the occurrence of the disease antenatally, so that all preparations may be made for treatment. Note the following points in the *family history* : (1) Few cases occur as a result of the first pregnancy ; (2) a history of stillbirths is twice as common in affected families as in the general population (Gordon), but repeated early abortions are of no significance except possibly in the very rare cases of $\alpha$ or $\beta$ incompatibility ; (3) a history of neonatal jaundice, anaemia or obscure neonatal death in siblings calls for investigation. Wherever the family history is suggestive, the blood of both parents should be sent for investigation during the pregnancy.

*Clinical Signs and Symptoms.*—The following signs in the new-born infant call for investigation for haemolytic disease : (1) Placenta enlarged and pale or the fluid yellow ; (2) Jaundice appearing in the first two days of life, particularly if it deepens rapidly and is accompanied by much lethargy and urine deeply coloured. Stools are usually normal, but if the jaundice persists they may be bile-free and pale for short periods owing to plugging of the bile ducts in the liver with large bile thrombi ; (3) Anaemia, present at birth or developing during the first four weeks.

Oedema is common in the new-born in a variety of conditions, particularly atelectasis, and therefore not of significance unless accompanying jaundice or anaemia. Haemorrhages are common in the new-born owing to low prothrombin, but in haemolytic disease skin and pulmonary haemorrhages are particularly liable to occur rather than gastro-intestinal tract haemorrhage. Neck rigidity, spasticity or actual convulsions, lethargy, occur in deeply jaundiced infants owing to kernicterus. Enlargement of spleen is not common in the early stages, except in hydrops foetalis or severe anaemia, but usually develops in untreated cases during the later stages.

*Diagnosis.*—Table I gives average figures for the first eight weeks of life.

TABLE I

NORMAL NEONATAL BLOOD COUNT

| | Birth | 4th Day | 10th Day | 14th Day | 8 Weeks |
|---|---|---|---|---|---|
| Hæmoglobin per cent. | 130–140 | 150–160 | 120–130 | 100 | 85 |
| R.B.C. (millions per c.mm.) | $5\frac{1}{2}$ | 5–6 | 5 | $4\frac{1}{2}$ | $4\frac{1}{2}$ |
| W.B.C. thousands per c.mm.) | 20–40 | — | — | 10–20 | — |
| Erythroblasts, per cent. | 0–10 | 0 | 0 | 0 | 0 |
| Reticulocytes, per cent. | 3 | 4–6 | 0.8–1.5 | $>$ 1 | 0 |

In haemolytic disease changes in the blood count vary with the clinical type. In cases mainly characterized by jaundice there is usually little change in haemoglobin or red cell count for the first few days, after which a steady fall tends to occur if the infant survives ; haemoglobin as low as 100 per cent. on the fourth to fifth day of life should arouse definite suspicion of abnormal haemolysis, and a daily count may be necessary to reveal the presence of developing anaemia. In hydropic infants very low levels of haemoglobin, often below 30 per cent., are found at birth, and low figures develop with great rapidity in cases mainly characterized by severe anaemia rather than jaundice. A large percentage of cases shows an abnormally high number of erythroblasts in the circulating blood at birth, which persists after the first twenty-four hours ; or, in some cases, there is a high reticulocytosis. Severe erythroblastosis usually indicates a grave prognosis.

Rhesus testing of the blood of mother and child should be performed at once, and in the case of a still-born infant, the blood of both parents should be examined. The diagnosis is confirmed by the finding of rhesus antibodies in the mother's blood and a positive Coombs's test given by the infant's red cells (1945).

*Treatment.*—The morbid processes of this disease take place with such rapidity that the urgency of investigation and treatment in these cases cannot be over-emphasized. There are two aims in treatment: first, to provide the infant with sufficient red blood cells to prevent anoxia of tissues and to tide it over the period until sufficient unaffected red cells can be produced; and secondly, to combat the antibody-antigen reaction in the tissues before irreparable damage to liver and brain has occurred.

All these infants are severely ill and require skilled team-work if good results are to be obtained, i.e. adequate nursing technique, medical supervision and pathological facilities. All affected babies are transfused promptly with blood which is unlikely to undergo rapid haemolysis in the infant's circulation, i.e. Rhesus negative (D-negative) Group O blood for the majority of cases; but, in the rarer cases of incompatibility due to other rhesus genotypes, blood of the mother's rhesus typing must be used. (Not, of course, the mother's blood, owing to the presence of antibodies.) Recently packed cell transfusions have been given in order to reduce the volume of fluid introduced. The size of the transfusion varies with the degree of anaemia, but usually 10–15 c.cm. of packed cells per pound of body weight are given at a rate of 5–8 drops per minute. Frequent haemoglobin estimations and Coombs's testing are done after transfusion, and further transfusions are given if the haemoglobin level falls below 50 per cent.

The most difficult problem in treatment is the severely jaundiced baby without anaemia. Theoretically, benefit should be obtained by replacement transfusion, and good results have been reported in a small series (Wallerstein, 1947). The technical difficulties are considerable, and this procedure would not be effective in combating the tissue reactions which are believed to occur before birth. We have, therefore, transfused all affected babies, irrespective of anaemia and have noted considerable clinical improvement in those with severe jaundice only.

Witebsky and Anderson (1942) showed that breast milk might contain the rhesus antibody and that breast-fed children might fail to respond to treatment. In our series none of the breast-fed infants showed any delay in reaction to treatment or any increased severity of symptoms. During the past year breast milk has been tested in each case and no rhesus antibody has yet been demonstrated. It is therefore our practice to breast-feed all possible infants in view of the increased liability to infection and digestive disturbances in the artificially-fed infant, particularly those born in institutions. (*Report on Breast-feeding of Infants*, 1944.)

If the view is held that severe damage to the foetal liver and other tissues may occur *in utero* during the last weeks of pregnancy as a result of these antibody reactions, premature delivery and rapid transfusion treatment might offer some hope of a living, healthy child to parents who give a bad history of still-births or neonatal deaths from this cause. The success of such treatment would obviously depend on the choice of time for delivery (which might have to be by Caesarean section) and the provision of good facilities for the nursing care of the premature child, such as are only likely to be provided in a special premature baby unit. We have selected a small series of cases for treatment by premature Caesarean section at Southmead Hospital, with encouraging results. The cases were selected on the grounds of bad family history and the presence of a rising rhesus-titre in the maternal blood ; most of the cases being delivered at the 34th–36th week of pregnancy.

*Results of Treatment.*—Arrangements were made for rhesus-testing by the Army Blood Transfusion Service in Bristol in 1943, and continued by the South-West Regional Transfusion Service. Fifty-four definite cases of Haemolytic Disease have been investigated and treated in Bristol since the end of 1943. Fifty babies were treated by transfusion with thirty-seven survivors, giving a mortality rate of 26 per cent. Of the four cases not transfused but definitely diagnosed, one was a mongol first seen at three weeks old, jaundiced, anaemic and moribund ; one child died within a few hours of birth, before transfusion could be started ; one had severe hydrops foetalis and was moribund when first seen ; and another, very early in the series, had marked jaundice and physical signs of kernicterus, but no anaemia, and decision was made not to transfuse, the baby dying at seventy-two hours.

TABLE II

RESULTS OF TREATMENT

| | |
|---|---|
| Total cases investigated .. | 54 |
| Treated by Transfusion .. | 50 |
| Died .. .. .. .. | 13 = 26 per cent. |

Twenty-eight patients received one transfusion each, and only two needed as many as three transfusions. Nineteen of the survivors were fully breast-fed for the normal period of lactation, four babies were breast-fed for three to four months, and eleven for two weeks or less. Two cases developed temporary obstructive jaundice before discharge from hospital with bile-free stools, which subsequently became normal again. Caesarean section was performed on seven patients, resulting in six living children, two of whom survived.

The causes of death in the series are of interest. Two cases were diagnosed too late for treatment to be of any avail; haemorrhage accounted for death in five cases, and kernicterus, associated with intense jaundice and presumed liver insufficiency, was the only cause of death found in two cases; broncho-pneumonia and atelectasis accounted for four deaths.

Severe sequelae of kernicterus, mental defect, hepatic cirrhosis, green teeth and cystic bones have been frequently stressed in the past. In order to determine how far the intensive treatment outlined is justified an attempt has been made to follow up all the survivors of this series. Thirty-four babies have been examined by us during April, 1947. Of the remaining three, one died of broncho-pneumonia in another hospital, aged six months, and at post-mortem no sequelae of haemolytic disease were found; two have left the district and all efforts to trace them have so far failed.

No case had any enlargement of liver or spleen or detectable abnormality of bones. Five patients had definitely green teeth, the discoloration affecting all the teeth erupted at the time of examination. One patient had an ankylosed hip as a result of staphylococcal arthritis occurring during the neonatal period. One child had slight foot-drop which occurred following a tibial marrow blood transfusion for his haemolytic disease (this was the only tibial transfusion given). One child had definite mild hydrocephalus and spasticity of legs, was late in talking and walking and appears to be mentally deficient. With the exception of this one case, all the children had progressed well physically and mentally and appeared up to or above average standards. The two patients delivered prematurely by Caesarean section had made exceptionally good progress.

*Conclusions.*—This very important work is still in the experimental stage, and although the mortality rate has been reduced from 50–60 per cent. to 26 per cent., it could be reduced still further by better antenatal diagnosis. Premature delivery has as yet only been tried in a few cases, but the results are encouraging and show that some healthy children may be saved. A special plea must be made that as many cases as possible should be investigated and treated at research centres such as Bristol, because, although the disease is not as rare as previously supposed, it is still difficult to collect enough cases to produce any significant statistics for results of treatment. Also, these infants are all extremely ill and the best results are likely to be obtained if they can be delivered and cared for in special units under the most suitable conditions.

We desire to acknowledge with gratitude the co-operation of all the obstetric teams of Bristol Hospitals; and, also, valuable help given by Dr. Hackett, of the South-West Regional Blood Transfusion Service, in the "follow-up" of cases.

### REFERENCES.

1. Diamond, L. K., Blackfan, K. D. and Baty, J. M., 1932. *Journal of Paediatrics*, 1, 269.
2. Hampson, 1928. *Guy's Hospital Reports*, lxxviii, 199.
3. Dienst, A., 1908. *Archives f. Gynäk*, 86, 314.
4. Ottenberg, R., 1923. *Journal of American Medical Association*, 81, 295.
5. Hawksley, J. C. and Lightwood, R., 1934. *Quarterly Journal of Medicine*, 3, 159.
6. Wiener, A. S. and Peters, H. R., 1940. *Annals of Internal Medicine*, xiii, 2306.
7. Levine, P., Katzin, E. M. and Burnham, L., 1941. *Journal of the American Medical Association*, cxvi, 825.
8. Baar, H. S., 1945. *Austrian Medical Bulletin*, special issue, p.1.
9. Gordon, R. R., 1946. *Archives of Disease in Childhood*, 21, 225.
10. Coombs, R. R. A., Mourant, A. E., Race, R. R., 1945. *British Journal of Experimental Pathology*, 26, 255.
11. Wallerstein, H., 1947. *American Journal of Diseases of Children*, 73, 1, 19.
12. Witebsky, E., Anderson, G. W. and Heide, A., 1942. *Proceedings of the Society of Experimental Biology and Medicine*, 49, 179.
13. *Report on the Breast-feeding of Infants*, 1944. H.M. Stationery Office, London.

